# Supplementary material for: Asymmetric Synthesis of Quaternary Hydantoins via a Palladium-Catalyzed Aza-Heck Cyclization
Source: J Am Chem Soc. 2025 Nov 14;147(49):44692–8. doi: 10.1021/jacs.5c16022 (PMC12703750; doi:10.1021/jacs.5c16022)
Supplement: Supplementary file 2 [file ja5c16022_si_002.zip › All NMR FID Files/S8/S8_AllNMR/1/pdata/1/email_EM02-248-1_1_1.pdf]

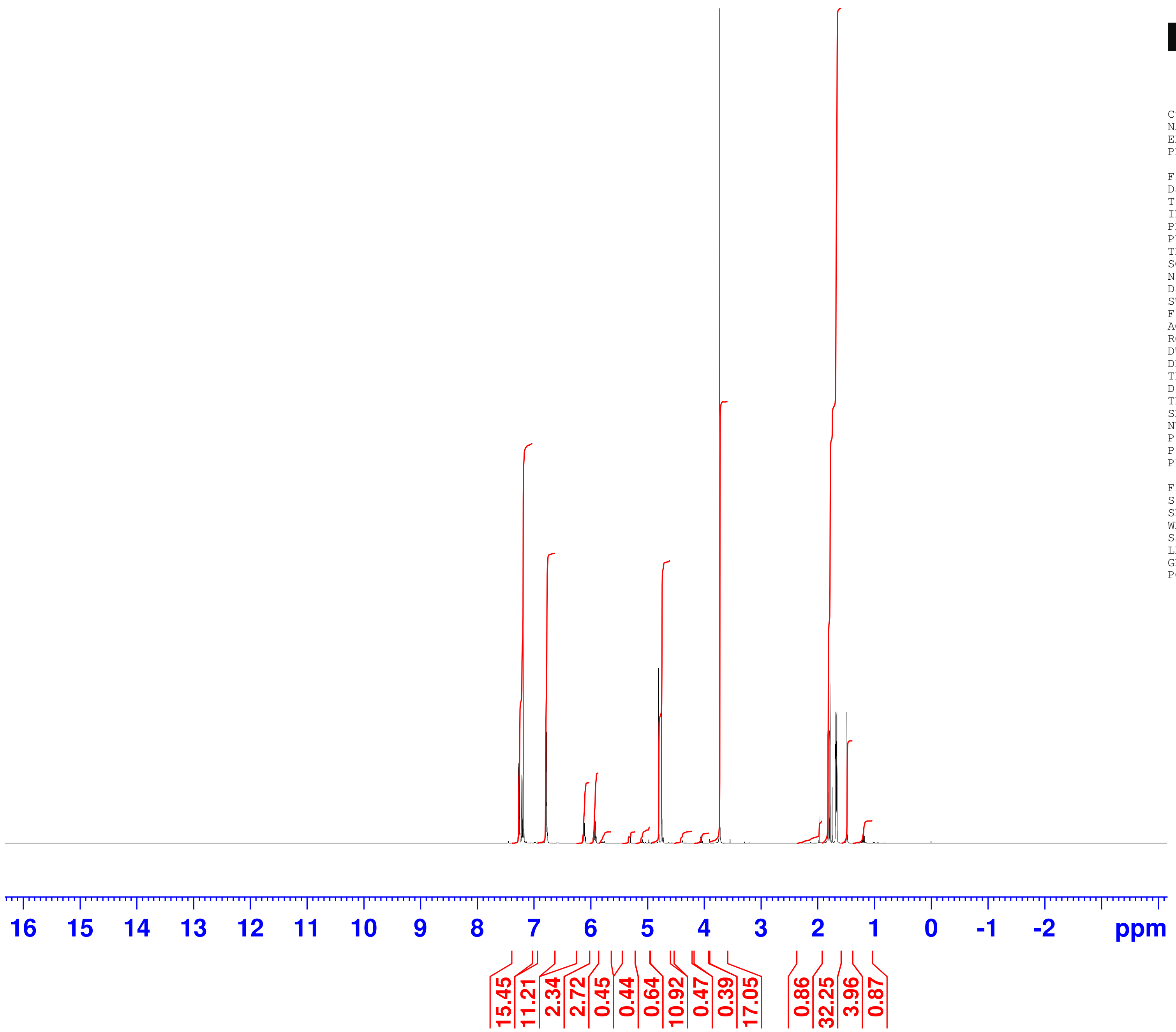

Current Data Parameters
NAME EM02-248-1
EXPNO 1
PROCNO 1

F2 - Acquisition Parameters
Date_ 20220614
Time 21.38 h
INSTRUM Avance Neo 400 U of DE Brown Lab
PROBHD Z105996_0006 (
PULPROG zg30
TD 65536
SOLVENT CDCl3
NS 16
DS 2
SWH 8196.722 Hz
FIDRES 0.250144 Hz
AQ 3.9976959 sec
RG 16.9271
DW 61.000 usec
DE 12.69 usec
TE 298.0 K
D1 1.00000000 sec
TD0 1
SFO1 400.1324708 MHz
NUC1 1H
P0 5.00 usec
P1 15.00 usec
PLW1 3.26480007 W

F2 - Processing parameters
SI 65536
SF 400.1300376 MHz
WDW EM
SSB 0
LB 0.30 Hz
GB 0
PC 1.00
